# 1313. Determinants of Prosthetic Joint Infection According to Time Following Total Knee Arthroplasty: A Retrospective Cohort Study Among US Veterans

**DOI:** 10.1093/ofid/ofad500.1152

**Published:** 2023-11-27

**Authors:** Erica J Weinstein, Vincent Lo Re, Alisa Stephens-Shields, Craig Newcomb, Randi Silibovsky, Charles Nelson, Judith O’Donnell, Laurel Glaser, Evelyn Hsieh, Jennifer Hanberg, Kathleen Akgün, Janet Tate, Joseph King

**Affiliations:** Perelman School of Medicine at the University of Pennsylvania, Philadelphia, Pennsylvania; University of Pennsylvania, Philadelphia, PA; Perelman School of Medicine at the University of Pennsylvania, Philadelphia, Pennsylvania; Perelman School of Medicine at the University of Pennsylvania, Philadelphia, Pennsylvania; Perelman School of Medicine at the University of Pennsylvania, Philadelphia, Pennsylvania; Perelman School of Medicine at the University of Pennsylvania, Philadelphia, Pennsylvania; Perelman School of Medicine at the University of Pennsylvania, Philadelphia, Pennsylvania; University of Pennsylvania, Philadelphia, PA; Yale University School of Medicine and VA Connecticut Health System, West Haven, Connecticut; Massachusetts General Hospital, Boston, Massachusetts; Yale University School of Medicine and VA Connecticut Health System, West Haven, Connecticut; Yale University School of Medicine and VA Connecticut Health System, West Haven, Connecticut; Yale University School of Medicine and VA Connecticut Health System, West Haven, Connecticut

## Abstract

**Background:**

Despite the clinical impact of prosthetic joint infections (PJIs), the factors associated with these infections remain unclear. We sought to determine patient and surgical determinants of PJIs occurring ≤ 3 months (early period), > 3 and ≤ 12 months (delayed period), and > 12 months (late period) after primary total knee arthroplasty (TKA).

**Methods:**

We conducted a retrospective study of patients in the Veterans Aging Cohort Study- National Cohort who underwent elective primary TKA in the Veterans Health Administration (VA) between October 1, 1999 to September 30, 2019 and had data available in the VA Surgical Quality Improvement Program (VASQIP). The primary outcome was incident hospitalization with PJI. A piecewise exponential parametric survival model fit through Poisson regression was used to estimate incidence rate ratios (IRRs) with 95% confidence intervals (CIs) of early, delayed, and late PJI associated with demographic, clinical, and peri-operative factors after accounting for clustering by VA center and adjustment for year of TKA (categorized in 5-year periods).

**Results:**

A total of 67,915 patients with TKAs were included in the cohort, of whom 1,308 developed an incident PJI (516 early; 292 delayed; 500 late). The median age was 65 (interquartile range [IQR], 60-71) years, and the majority were male (94.7%). Hypertension (86.4%), obesity (64.0%), and diabetes mellitus (38.2%) were common. In multivariable analysis, early PJI was associated with HCV infection, autoimmune inflammatory arthritis, urban location, and prolonged operative time (**Table 1)**. Delayed PJI was associated with alcohol use disorder, anemia, HCV infection, autoimmune inflammatory arthritis, and prolonged operative time. Late PJI was associated with alcohol use disorder, anemia, diabetes mellitus, HCV infection, and autoimmune inflammatory arthritis.

Adjusted incidence rate ratios of early (n=516 events), delayed (n=292 events) and late (n=500 events) prosthetic joint infections after primary total knee arthroplasty associated with demographic, baseline clinical and peri-operative factors among patients (n=67,915).

**Figure:**
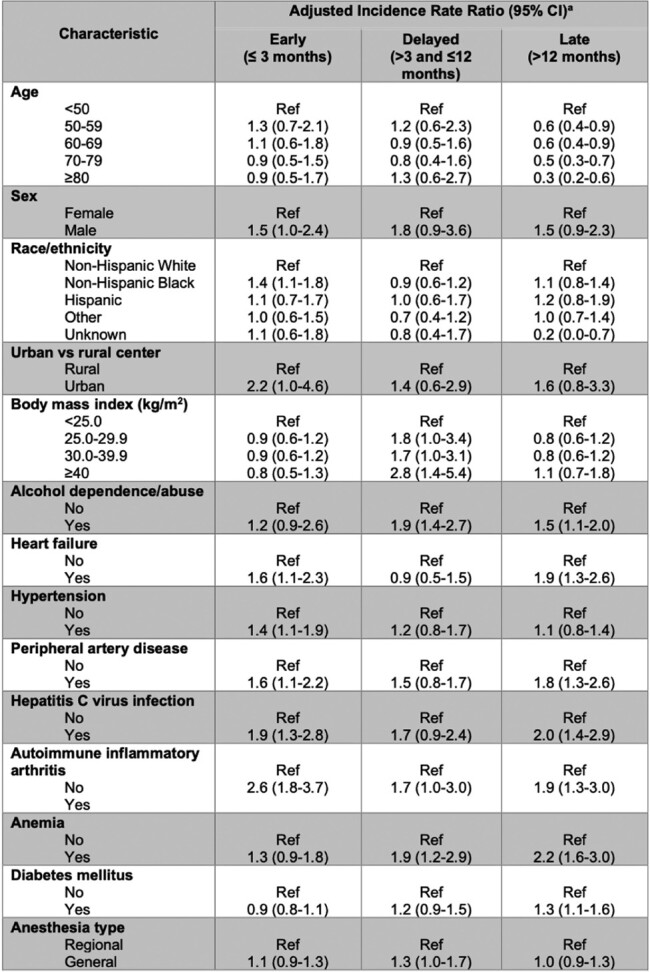

Abbreviations: No., number; PJI, prosthetic joint infection; pmonths, person months; IRR, incidence rate ratio, TKA, total knee arthroplasty. aIncidence rate ratios for each variable were adjusted for all other variables as well as by VA site and year of TKA (categorized in 5-year periods from 1999-2019).

**Conclusion:**

Factors contributing to impaired wound healing or tissue hypoxia were associated with early PJI (autoimmune inflammatory arthritis, prolonged operative time), whereas those associated with increased risk of local skin/soft tissue and hematogenous infections (alcohol use, diabetes, autoimmune inflammatory arthritis) over the life of the joint were associated with delayed or late infections.

**Disclosures:**

**Alisa Stephens-Shields, PhD**, Gilead Sciences: Advisor/Consultant **Charles Nelson, MD**, Stryker: Advisor/Consultant|Zimmer-Biomet: Advisor/Consultant|Zimmer-Biomet: Grant/Research Support

